# Negative pressure wound therapy versus usual care for Surgical Wounds Healing by Secondary Intention (SWHSI trial): study protocol for a randomised controlled pilot trial

**DOI:** 10.1186/s13063-016-1661-1

**Published:** 2016-11-08

**Authors:** Catherine Arundel, Hannah Buckley, Emma Clarke, Nicky Cullum, Stephen Dixon, Jo Dumville, Caroline Fairhurst, Anna Firth, Eileen Henderson, Karen Lamb, Elizabeth McGinnis, Angela Oswald, Pedro Saramago Goncalves, Marta Soares, Nikki Stubbs, David Torgerson, Ian Chetter

**Affiliations:** 1 York Trials Unit, Department of Health Sciences, University of York, York, YO10 5DD UK; 2 Hull and East Yorkshire Hospitals NHS Trust, Hull, UK; 3 School of Nursing, Midwifery and Social Work, The University of Manchester, Manchester, UK; 4 Patient and Public Involvement Group Representative, Hull, UK; 5 Leeds Community Healthcare NHS Trust, Leeds, UK; 6 Leeds Teaching Hospitals NHS Trust, Leeds, UK; 7 Centre for Health Economics, University of York, York, UK; 8 Hull York Medical School, York, UK

**Keywords:** Surgical wounds, Negative pressure wound therapy, Secondary intention, Healing, Randomised controlled trial

## Abstract

**Background:**

Most incisions following surgery heal by primary intention, with the edges of the wound apposed with sutures or clips. However, some wounds may break open or be left to heal from the bottom up (i.e. healing by secondary intention). Surgical Wounds Healing by Secondary Intention (SWHSI) are often more complex to manage, and require additional treatments during the course of healing. There is significant uncertainty regarding the best treatment for these complex wounds, with limited robust evidence regarding the clinical and cost-effectiveness of different dressings and treatments; one such treatment is Negative Pressure Wound Therapy (NPWT) which is frequently used in the management of SWHSI. Previous randomised controlled trials (RCTs) of NPWT have failed to recruit to time and target, thus we aimed to conduct a pilot RCT to assess the feasibility of conducting a future, full-scale RCT.

**Methods:**

This pilot RCT will test the methods and feasibility of recruiting, randomising, and retaining participants into a larger trial of NPWT verses usual care for patients with SWHSI. Participants will be randomised to receive either NPWT or usual care (no NPWT) and will be followed up for 3 months.

**Discussion:**

This study will provide a full assessment of methods for, and feasibility of, recruiting, randomising, and retaining patients with SWHSI in a trial of NPWT versus usual care. On the basis of this pilot trial, a full trial may be proposed in the future which will provide additional, robust evidence on the clinical and cost-effectiveness of NPWT in the management of SWHSI.

**Trial registration:**

Clinical Trial Registry: ISRCTN12761776, registered on 10 December 2015 – retrospective registration.

**Electronic supplementary material:**

The online version of this article (doi:10.1186/s13063-016-1661-1) contains supplementary material, which is available to authorized users.

## Background

A substantial number of surgical operations are conducted in the NHS each year, with most involving an incision [1]. The edges of these incisions are often held together whilst healing occurs (primary closure); however, many surgical wounds break open or are left open to heal (healing by secondary intention). Surgical Wounds Healing by Secondary Intention (SWHSI) present a significant management challenge as they may remain open for many months and/or require multiple, additional treatments (e.g. prolonged hospitalisation, reoperation, infection management) [2]. As a result, management of SWHSI presents a significant financial burden to the NHS and also impacts substantially on patients’ quality of life.

There is much research evidence available to guide the management of SWHSI; however, as NICE reflected in their 2012 guidelines [3], there is a need for robust, experimental evidence to assess the most clinical and cost-effective dressings and treatments for surgical site management. Negative Pressure Wound Therapy (NPWT) has become a widely used intervention for SWHSI, albeit more frequently within acute rather than community settings [4]. NPWT devices apply negative pressure to a wound via a dressing which theoretically promotes wound healing by removing exudate and reducing infections [5]. The device is generally only used for part of the SWHSI treatment pathway rather than to the point of healing.

A Cochrane Review in 2015 [6] specifically investigated NPWT as a treatment for SWHSI; however, the review found only two trials eligible for inclusion. On the basis of this limited evidence and the fact that NPWT is widely used in the NHS to treat SWHSI, there is an urgent need to assess the effectiveness of this intervention in this specific patient population.

Previous trials of NPWT have struggled to recruit [7, 8] and so it would seem to be essential, before embarking on a full RCT, to assess the feasibility of conducting such research. Our study, therefore, seeks to ascertain the feasibility of conducting a full RCT in this area, specifically investigating the appropriate methods to use to collect meaningful and worthwhile data.

## Methods

### Design

This study is a pilot RCT conducted in three centres to test the methods and feasibility of a full RCT of NPWT compared with usual care (no NPWT) for SWHSI. Informed consent will be obtained from each patient prior to randomisation into the study. Eligible patients will be individually randomised to one of two treatment arms: (1) NPWT or (2) usual care (no NPWT).

The key objectives of this trial are to determine the methods and feasibility of conducting a larger RCT in this area. Specifically, this pilot trial will assess:

1. Recruitment rate including willingness of participants to be randomised and whether recruitment is influenced by wound location or other factors, e.g. associated surgical speciality
2. Clinician willingness and ability to recruit and randomise participants
3. Testing of inclusion and exclusion criteria
4. Fitness for purpose of data collection methods including across and between care settings
5. Ability of sites and clinicians to supply NPWT to intervention participants in a timely fashion, irrespective of care setting, and to assess any training requirements
6. Ability of community staff to manage participants randomised to NPWT
7. Suitability of method of outcome ascertainment
8. Adequacy of duration of follow-up
9. Rates of withdrawal from treatment, response rates to questionnaires, attrition from the trial, and likely rates of missing data for outcomes
10. Assessment of feasibility of blinding outcome assessors to treatment allocation
11. Acceptability of trial documentation to nurses collecting study data in addition to treating patients
12. The primary outcome for this pilot trial will be time to complete wound healing (full wound epithelialisation). However, this pilot study is not looking to detect a treatment effect but to determine the ability to recruit to, and collect high-quality data in, a full RCT. Secondary outcomes and methods of data collection are summarised in Table 1.

**Table 1:** Secondary outcome measures

Secondary outcome	Measured by/using
Number of patients screened	Pre-Trial Screening Assessment Form
Number of patients eligible/ineligible	Pre-Trial Screening Assessment Form
Appropriateness of eligibility criteria and reasons for ineligibility	Pre-Trial Screening Assessment Form
Proportion of eligible patients consenting to participate (and whether type of wound/surgical speciality impacts on this)	Consent Form and Pre-Trial Screening Assessment Form
Time between randomisation and treatment start	Phase 1 Start of Treatment Form
Proportion of patients receiving randomised treatment within 48 h	Phase 1 Start of Treatment Form
Duration of Negative Pressure Wound Therapy (NPWT)	Phase 1 Start of Treatment Form and End of Phase 1 Treatment Form
Factors affecting timely delivery of randomised treatment	Non-immediate Use of NPWT Form
Wound dimensions	Assessment of Weekly Participant Events Form
Quality and completeness of data collected for date of healing	Assessment of Weekly Participant Events Form and Blinded Outcome Assessment Form
Patient-reported health-related quality of life	The Short Form (12) Health Survey (SF-12) [13] EuroQoL (EQ-5D) [14]
Cointerventions used and rate of treatment change	Investigator Baseline Case Report Form (CRF), Assessment of Weekly Participant Events Form, Phase 1 Start of Treatment Form, Phase 1 End of Treatment Form
Significant events (e.g. rehospitalisation, infection, reoperation)	Assessment of Weekly Participant Events Form
Adverse events	Adverse Event Form, Serious Adverse Event Form, Adverse Event/Serious Adverse Event Follow-up Form
Response rates	Proportion of participant self-report measures returned
Withdrawal rates	Change of Status Form
Treatment change including change from NPWT for intervention participants, reasons for changes and changes to NPWT	Surgical Wounds Dressing Change Form, End of Phase 1 Treatment Form and Assessment of Weekly Participant Events Form
Resource use	Resource Use Questionnaire (3-month Participant CRF)
Missing data rates	Missing responses
Healing rates	Assessment of Weekly Participant Events Form, Blinded Outcome Assessment Form
Blinded outcome assessment	Blinded Outcome Assessment Form
Participant opinion on trial participation	Likert Scales (3-month Participant CRF)
Self-reported wound pain	Weekly text messaging Visual Analogue Scales (baseline, 2-week, 1-month and 3-month Participant CRFs) Brief Pain Inventory (BPI) (baseline, 2-week, 1-month and 3-month Participant CRFs) [15]
Wound progress	Photographs Wound tracings Depth measurements

**Fig. 1:**
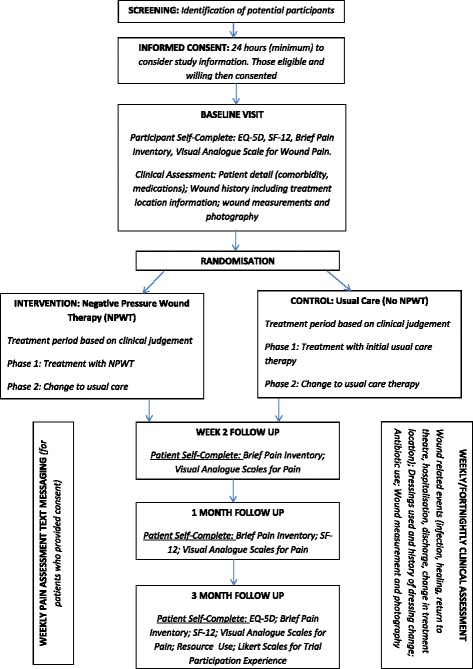
Participant flowchart and schedule of activity. Outlining participant flow through the study and outcome measurement completion time points

Participants will be followed up every 1–2 weeks, depending on feasibility, for clinical outcome assessment. Participant-completed questionnaires will be completed at baseline, 2 weeks, 1 month, and 3 months post randomisation. Participants will be followed from randomisation to trial exit, which is deemed to be 3 months after randomisation, or loss to follow-up or death (if before this time). Participant flow through the trial and the schedule of study activities are displayed in Fig. 1.

### Inclusion and exclusion criteria

#### Inclusion

Our target population will be patients aged 18 years and over who are able to give full informed consent and who: are receiving care from either Hull and East Yorkshire Hospitals NHS Trust, Leeds Teaching Hospitals NHS Trust or Leeds Community Healthcare NHS Trust; have a SWHSI which could be reasonably treated with NPWT or wound dressings; have a SWHSI which is considered ready for NPWT (i.e. minimum 80 % viable tissue or thin layer of slough requiring no further debridement); and are receiving adequate nutrition (as assessed by the senior nurse responsible for nursing care).

#### Exclusion

We will exclude patients who: are unable to give informed consent; have limited life expectancy (e.g. are undergoing end-stage palliative care); have an active systemic infection; have already received NPWT on their current SWHSI, are currently receiving NPWT or received NPWT whilst in theatre for the surgery resulting in their SWHSI; have inadequate haemostasis or are at risk of bleeding; have chronic wounds such as pressure or foot ulcers which are nonsurgical in origin but which have been surgically debrided (we regard these patients as a distinctly different subgroup); are unwilling to have wound photographs taken; or are currently or have previously participated in a research study within the last 4 weeks.

Patients will also be excluded if they have any of the following wound characteristics present: unclear undermining in the wound cavity precluding use of NPWT; necrotic tissue or eschar; malignant tissue; exposed blood vessels and/or organs, anastomotic sites, and/or nerves (including cases where abdominal fascia is open); or located where a vacuum seal cannot be obtained (in the opinion of the treating clinician).

### Recruitment

Patients will be recruited from both acute and community settings within Hull and Leeds; however, we anticipate that most participants will be recruited from acute settings. The pilot trial will be preceded by promotion of the study with surgeons and nurses in both settings.

All patients who experience a SWHSI (at any point following surgery) will be screened for trial eligibility by their clinical care team. Potential participants will then be approached with further details of the study by surgical or nursing staff (clinical or research) during ward rounds, routine care or home visits, depending on patient circumstance.

Potential participants will be provided with a patient information sheet and will be given at least 24 h to consider their involvement in this research. Patients will then have the opportunity to discuss the study with the research team prior to completing the study consent form.

We aim to randomise 50 patients for this pilot trial over a 7-month period. As evidenced by Edwards et al. in 2002 [9], nonconditional, monetary incentives are shown to double response rates to postal questionnaires; participants will, therefore, be sent a £5 unconditional cash token with their final questionnaire (at 3 months post randomisation). Participants who consent to receiving weekly text messages, and provide a mobile telephone number to facilitate this, will also be reimbursed £5 to cover any costs incurred in responding to weekly pain assessment text messages.

### Randomisation

Patients will be randomised into one of two arms: (1) NPWT or (2) usual care (no NPWT) on a 1:1 basis. To remove the potential for selection bias, allocations will be concealed through use of a central randomisation service implemented by an independent member of staff at the York Trials Unit, University of York. Study sites will contact a remote telephone service to provide details of the participant and to receive details of the treatment allocation. Randomisation will be conducted using a pregenerated sequence of random permuted blocks with stratification by wound area (<28 cm^2^, ≥28 cm^2^).

Due to the nature of the intervention it is not possible to blind participants or health care professionals to trial treatment. Feasibility of blinded outcome assessment of healing will, however, be assessed as part of this trial. Assessors blind to trial treatment allocation will be provided with copies of wound photographs and will be asked to confirm whether (1) they deem the wound to be healed (full wound epithelialisation), and (2) they believe they know the allocated treatment allocation.

### Sample size

As this is a pilot trial, and the primary objective is to determine measures of feasibility and acceptability rather than to detect a treatment effect, a formal sample size calculation has not been conducted. We will, however, aim to randomise approximately 50 patients as a means to assess recruitment, randomisation, and retention of participants.

### Interventions

#### Experimental group: Negative Pressure Wound Therapy

There are several NPWT systems available within the NHS; however, for the purposes of this trial we originally permitted the use of the following CE-marked products currently used in acute and community settings in Hull and Leeds: (1) V.A.C.® (KCI), and (2) Renasys® (Smith and Nephew). A further device (PICO® – Smith and Nephew) has subsequently been added as a trial device in a recent protocol amendment.

NPWT treatments consist of a vacuum pump into which a disposable plastic canister is placed to enable wound exudate to be collected. The canister is attached to pressure resistant tubing to create an airtight seal. The wound to be treated is filled with a suitable dressing (foam or gauze) along with a nonadherent layer, if required, to protect blood vessels or organs and/or to prevent dressing adherence.

In this trial, the choice of machine and duration of NPWT will be decided by the treating health professional in conjunction with the participant and nurse. Pressure cycles and dressing change frequency will be completed as per standard practice and recorded. The only stipulation with regards NPWT use is that this must be clinically appropriate. When not being treated with NPWT, participants in this arm of the trial will be treated as per usual care.

#### Comparator group: Usual Care (no NPWT)

The control group participants will receive usual care. This is likely to be wound dressings which will be changed every 1–3 days, or sometimes less frequently, as determined by the treating surgeon or nurse in line with standard practice. The trial protocol does not stipulate the type of dressings which should be used as part of usual care as there is no evidence to suggest that any one dressing is more clinically or cost-effective than another. Control dressings are, therefore, selected by the clinician on the basis of the dressing most appropriate for the patient. The types of primary and secondary dressings used, and the frequency of dressing change, will be recorded throughout the trial.

### Statistical analysis

A full statistical analysis plan for primary and secondary analyses will be generated prior to completion of recruitment for this trial. The Trial Management Group and the Data Monitoring and Ethics Committee (DMEC) will review this prior to commencement of outcome analysis. During the trial regular reports will be prepared for the DMEC with regards data quality and safety.

The analyses will be conducted following the principles of intention-to-treat. All outcomes will be summarised using descriptive statistics overall and by trial group.

The primary clinical outcome of time to healing will be presented using Kaplan-Meier curves for interest only, recognising that the study is not powered to detect clinically important treatment effects. This analysis will be conducted using blinded outcome assessment dates of healing if possible. A Cox proportional hazards regression analysis will be conducted, subject to sufficient data, to investigate the inclusion of additional covariates shown to be important in an earlier SWHSI cohort (e.g. contamination level of surgery, wound infection), along with the stratification factor (baseline wound size) used in the randomisation for this trial. The impact of SWHSI history, location of SWHSI on the body and infection at any time during follow-up (as a time dependent covariate) will also be explored.

Cost-effectiveness will be explored by calculating a mean total cost per trial arm using resource use and relevant unit costs for each participant. Should sufficient data be available, EuroQoL 5 dimensions (EQ-5D) questionnaire data will be used to calculate a mean quality-adjusted life year (QALY) for each trial arm.

### Patient and Public Involvement

Patient and Public Involvement (PPI) comes from a patient user group comprising 10 members who have contributed to the design and conduct of the study. This has included input on the outcomes collected, study documentation design and wording, and will include input into planned dissemination of research findings. The group members are patients who have participated in an earlier SWHSI cohort study. PPI members will be appropriately reimbursed for their participation in line with INVOLVE guidelines [10].

### The Trial Steering and Data Monitoring and Ethics Committees

As this is a pilot trial, the study will be monitored closely by an in-house Trial Management Team who will meet on a monthly basis. A Trial Steering Committee will not be convened for this trial.

A Data Monitoring and Ethics Committee (DMEC) will be formed to routinely review data, participant safety, and protocol compliance. The committee will consist of independent members: a statistician; two clinicians; and a PPI representative who will meet, at minimum, once prior to study commencement and once during the study duration.

### Forecast execution dates

The set-up of the trial commenced on 8 October 2015, following Research Ethics Committee (REC) approval, and was completed in March 2016. Recruitment of participants commenced on 20 November 2015 and will be completed on 30 June 2016. Follow-up for the trial commenced in December 2015 and will continue until 30 September 2016. Data analysis will commence in October 2016. The final report associated with this programme grant will be submitted in February 2017.

### Protocol changes

Protocol amendments made since the start of this trial are detailed in Table 2.

**Table 2:** Protocol amendments made following commencement of the trial

Aspect of the trial	Amendment made
Wound measurements	Depth measurement will be conducted by swab or probe
Adverse events	Serious, related, and ongoing events will be followed up for 1 month after trial exit
	Clarification of definitions of unplanned and prolonged hospitalisation
Intervention/control	Frequency of surgical wound dressing changes will be recorded
	Addition of further trial device (PICO® – Smith and Nephew)
Study processes	Patients who have their reference wound amputated will continue to be followed up for participant-reported measures
Patient and Public Involvement (PPI)	Patients in the SWHSI trial will be approached at the end of their trial involvement, with regards involvement in a PPI group.

*SWHSI* surgical Wounds Healing by Secondary Intention

The study protocol has been written in accordance with the Consolidated Standards of Reporting Trials (CONSORT) statement [11] and the Standard Protocol Items: Recommendations for Interventional Trials (SPIRIT) checklist [12], as provided in Additional file 1.

## Discussion

The proposed trial will explore methods for, and feasibility of, completing a full RCT to assess clinical and cost-effectiveness of NPWT for treatment of SWHSI compared with usual care. As would be expected with a feasibility trial, some barriers have been identified which may impact on recruitment and time to completion of the pilot or larger RCT in the future.

### Engagement of sites

During the set-up of the trial, the research sites have worked closely with their clinical colleagues to promote the trial. Promotion included contact with specialist teams, letters to surgeons, and posters advertising the study to clinical staff. Despite these efforts engagement of surgical and clinical colleagues has been mixed which has resulted in slower than anticipated recruitment. Continued efforts at a local level have helped to alleviate the lack of engagement as the trial has progressed resulting in an improvement in recruitment rate. Substantial work during set-up, and use of additional strategies to engage clinical staff should, however, be explored ahead of commencement of a larger RCT.

### Governance issues

Governance delays arose in one NHS trust due to circumstances outside of the trial’s control. This subsequently delayed opening of two colocated study sites, which impacted upon recruitment rate in the early stages of the trial. Despite this reduced recruitment in the early stages of the trial, recruitment has subsequently increased in all trusts, with the study meeting the target monthly recruitment in the following 4 months.

## Trial status

At the time of submission the trial is open to recruitment.

## Additional file

Additional file 1: SPIRIT 2013 checklist: recommended items to address in a clinical trial protocol and related documents. (DOC 119 kb)

## Abbreviations

BPI: Brief Pain Inventory
DMEC: Data Monitoring and Ethics Committee
EQ-5D: EuroQol 5 dimensions
NHS: National Health Service
NPWT: Negative Pressure Wound Therapy
QALY: Quality-adjusted life year
RCT: Randomised controlled trial
SF-12: Short Form 12
SWHSI: Surgical Wounds Healing by Secondary Intention

## References

[1] Key statistics on the NHS – NHS Confederation [Internet]. Available from: http://www.nhsconfed.org/resources/key-statistics-on-the-nhs. Accessed 11 June 2016.

[2] Mees J, Mardin WA, Senninger N, Bruewer M, Palmes D, Mees ST (2012). Treatment options for postoperatively infected abdominal wall wounds healing by secondary intention. Langenbecks Arch Surg.

[3] Surgical site infection – Guidance and guidelines – NICE [Internet]. Available from: https://www.nice.org.uk/guidance/cg74. Accessed 12 June 2016.

[4] Gregor S, Maegele M, Sauerland S, Krahn JF, Peinemann F, Lange S (2008). Negative pressure wound therapy: a vacuum of evidence?. Arch Surg.

[5] Science behind wound therapy [Internet]. Available from: http://www.kci1.com/KCI1/sciencebehindwoundtherapy. Accessed 11 June 2016.

[6] Dumville JC, Owens GL, Crosbie EJ, Peinemann F, Liu Z. Negative pressure wound therapy for treating surgical wounds healing by secondary intention. Cochrane Database Syst Rev 2015, Issue 6. Art. No.: CD011278. DOI: 10.1002/14651858.CD011278.pub2. 10.1002/14651858.CD011278.pub2 PMC10960640 26042534

[7] Ashby RL, Dumville JC, Soares MO, McGinnis E, Stubbs N, Torgerson DJ, Cullum N (2012). A pilot randomised controlled trial of negative pressure wound therapy to treat grade III/IV pressure ulcers [ISRCTN69032034]. Trials.

[8] Negative Pressure Wound Therapy for the Treatment of Chronic Pressure Wounds – Full Text View – ClinicalTrials.gov [Internet]. Available from: http://clinicaltrials.gov/ct2/show/NCT00691821. Accessed 11 June 2016.

[9] Edwards P, Roberts I, Clarke M, DiGuiseppi C, Pratap S, Wentz R, Kwan I (2002). Increasing response rates to postal questionnaires: systematic review. BMJ.

[10] Payment for involvement [Internet]. [Accessed 11.06.2016]. Available at: http://www.invo.org.uk/posttypepublication/payment-for-involvement/.

[11] Moher D, Schulz KF, Altman DG, CONSORT (2001). The CONSORT statement: revised recommendations for improving the quality of reports of parallel group randomized trials. BMC Med Res Methodol.

[12] Chan AW, Tetzlaff JM, Altman DG, Laupacis A, Gøtzsche PC, Krleža-Jerić K (2013). SPIRIT 2013 Statement: defining standard protocol items for clinical trials. Ann Intern Med.

[13] Ware JE, Kosinski M, Keller SD (1996). A 12-Item Short-Form Health Survey: construction of scales and preliminary tests of reliability and validity. Med Care.

[14] Brooks R, Rabin R, de Charro F (2003). The measurement and valuation of health status using EQ-5D: a European perspective.

[15] Cleeland CS, Ryan KM (1994). Pain assessment: global use of the Brief Pain Inventory. Ann Acad Med Singapore.

